# Comparison of the Effects of Posterior Cervical Fixation or Posterior Cervical Fixation Extending to the Upper Thoracic Region on Cervical Sagittal Alignment

**DOI:** 10.1111/os.14167

**Published:** 2024-07-23

**Authors:** Mustafa Kaya, Davut Ceylan, Tibet Kacira, Sabahattin Hiziroglu, Cigdem Erdin, Özlem Kitiki Kacira

**Affiliations:** ^1^ Department of Neurosurgery Sakarya University Faculty of Medicine Sakarya Turkey; ^2^ Department of Neurosurgery Sakarya University Training and Research Hospitaly Sakarya Turkey

**Keywords:** Cervical Alignment, Cervical Balance, Lateral Mass Screw, Pedicle Screw

## Abstract

**Objective:**

For degenerative diseases accompanied by cervical malalignment, the starting and ending points of fixation for better cervical sagittal alignment and clinical results are not as clear as the thoracolumbar region. In this study we aimed to compare the effects of posterior subaxial cervical fixation (PSCF), posterior cervical fixation extending to the upper thoracic region and posterior upper cervical fixation extending to the upper thoracic region on cervical sagittal alignment.

**Methods:**

Sixty‐three patients who underwent posterior cervical and cervical‐up thoracic fixation were retrospectively analyzed in a comparative study. The procedures that we performed from May 2019 to March 2022 on these 63 patients were: (1) C3‐C6 group—posterior subaxial cervical fixation; (2) Subaxial‐T2 group—posterior subaxial cervicothoracic fixation (PSCTF); (3) C2‐T2 upper thoracic posterior fixation group. The C3‐C6 group had 27 patients, Subaxial‐T2 group had 24, and C2‐T2 group had 12. We determined the minimum follow‐up period as 12 months. C0‐2, C2‐7 lordosis angle, sagittal vertical axis (SVA), C2 slope, C7 slope, T1 slope, cervical slope, neck slope, and thoracic inlet angle (TIA) measurements were made in three patient groups. Comparatively, cervical sagittal alignment was evaluated.

**Result:**

In the C2‐T2 group, a significant increase in C2‐C7 lordosis, decrease in C2 slope, and increase in TS‐CL were observed. Significant C2‐C7 lordosis decrease, C2 slope increase, and TS‐CL decrease were observed in the C3‐C6 group. A significant increase in C2‐C7 lordosis and a decrease in C2 slope were observed in the subaxial‐T2 group. No significant change was observed in the TS‐CL angle.

**Conclusion:**

In cervical degenerative disorders accompanied by cervical malalignment, we recommend the C2‐T2 fixation method, which provides the desired C2‐C7 lordosis, SVA within the normal range, and the best Neck Disability Index results.

## Graphical abstract

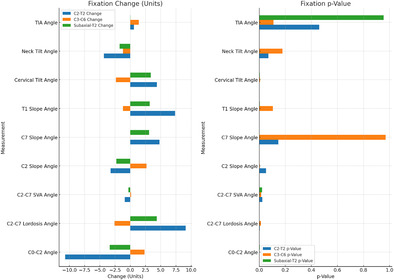

## Introduction

In degenerative diseases accompanied by cervical malalignment, fixation methods with lateral mass and pedicular screws from the posterior are widely used. When applying these methods, the starting and ending points of fixation for better cervical sagittal alignment and clinical results are not as clear as the thoracolumbar region.

Cervical spine alignment is critical for functional vision and sagittal balance of the spine.^1^ With developing technology, an increase in the development of cervical degenerative disorders and cervical kyphosis is observed due to people using devices such as mobile phones and computers for long periods of time while bending over in their daily business.^2^

Kyphosis of the cervical spine is the most common manifestation of cervical sagittal malalignment. As the kyphotic curve becomes more pronounced, it creases longitudinal cord tension by tethering the cervical spinal cord, leading to neuronal damage and demyelination.^3^, ^4^ Surgical treatment is usually performed to correct these radiological abnormalities and clinical symptoms. Posterior cervical stabilization is a frequently used surgical method in diseases affecting the cervical spine. The goals of surgery for cervical spine deformity are: list corrections of deformity, restoration of horizontal gaze, neural decompression, surgical correction and fusion to maintain sagittal alignment. Song et al. and Dru et al. reported that correcting kyphosis and providing adequate lordosis to ensure normal sagittal alignment has been shown to reduce neurological disorders.^5^, ^6^

Currently, surgical indications for correcting cervical alignment are not well defined, and there is no established standard to address the amount of correction desired. Although there are many publications showing quality of life and spinal alignment for the thoracolumbar region, there are fewer definitions for the cervical region.^3^

Cervical sagittal balance can affect the results of fusion, deformity correction, and degenerative cervical pathology operations. Posterior global misalignment after osteotomy for sagittal plane deformity can lead to sagittal overcorrection with posterior alignment, resulting in a compensatory loss of cervical lordosis.^7^

Evaluation of preoperative alignment is very important before performing posterior surgery to the cervical region. Recent studies have shown that, similar to the thoracolumbar spine, the severity of disability increases with positive sagittal malalignment after the cervical reconstruction surgery.^8^

In our study of cervical degenerative diseases, we determined three different surgical starting and ending points through two different surgical methods. The objectives of this study are threefold, to evaluate: (i) the effects of C3‐C6, Subaxial‐T2, and C2‐T2 posterior fixation on cervical alignment; (ii) these surgical methods clinically with visual analogue scale (VAS) and Neck Disability Index (NDI) measurements, and radiologically with C2 slop, C2‐C7 SVA, C2‐C7 lordosis and other aspects, and compare them; (iii) which surgical option we use in cervical degenerative disorders to obtain better cervical alignment, clinical and radiological results.

## Materials and Methods

### Description of Patients

The information and data of patients diagnosed with cervical stenosis and subaxial cervical trauma between May 2019 and March 2022 were examined retrospectively. Sakarya University Faculty of Medicine Deanship Non‐Interventional Ethics Committee has approved the retrospective study (E‐71522473‐050.04‐340191‐44). Preoperative lateral cervical radiographs, cervical computer tomography, and cervical magnetic resonance images of 76 patients diagnosed with cervical stenosis and trauma were examined and their diagnoses were checked. The inclusion criteria were: (1) patients with complete clinical and radiological data; (2) patients with cervical degenerative disorders confirmed by preoperative radiological and clinical data; (3) patients who underwent only a posterior surgical approach.

The exclusion criteria were: (1) patients who had cervical disc herniation surgery; (2) patients with less than three levels of instruments placed; (3) patients with occiput and C1 fusions; (4) patients diagnosed with tumor and infection.

Finally, 27 patients in C3‐C6 lateral mass group, 24 patients in Subaxial‐T2 group, and 12 patients in C2‐T2 group (63 patients total) with at least 12 months follow‐up were included in the study.

### Radiographic Analysis

It was evaluated with preoperative, postoperative, 3rd, 6th, and 12th month cervical radiographs. We had to take and evaluate scoliosis radiography in patients in whom we had difficulty imaging the lower cervical region. All radiographs were taken while standing in a neutral position and looking forward.

#### C0‐C2

It is the measurement of the angle between the McRae line and the C2 lower endplate with the Cobb method.

#### C2‐C7 Lordosis Angle

Lines are drawn along the lower endplates of C2 and C7, then additional lines are drawn perpendicular to the two lines. The angle formed by the vertical lines is the CL angle.

#### C2 Slop

Angle between the horizontal line and the line passing through the C2 lower endplate.

#### C7 Slop

Angle between the horizontal line and the line passing through the C7 lower endplate.

#### C2‐C7 SVA

The distance between the C2 plumb line and the posterior corner of the C7 corpus.

#### T1 Slop

The angle between the line drawn from the upper endplate of the T1 vertebra and the line drawn parallel to the ground from the same point.

#### Cervical Tilt

The angle between the line connecting the T1 upper endplate midpoint with the top of dens and the line drawn 90° to the T1 upper endplate midpoint.

#### Neck Tilt

It is defined as the angle between the line drawn from the highest point of the sternum perpendicular to the ground and the line drawn from the same point to the midpoint of the T1 upper endplate.

#### TIA

It is defined as the angle between the line drawn perpendicular to the T1 upper endplate midpoint and the line drawn from the upper border of the sternum to this point.

### Surgical Procedures

3D cervical CT, cervical and scoliosis radiographs were taken in all patients before surgery. The pedicle, mass, vertebral artery and corpus structures were examined. All patients were placed in a prone position with a skull clamp. After midline incision, spinous processes, laminas and facets were exposed by subperiosteal dissection. Magerl technique was used for lateral mass screws. In this technique, the entry point is 1 mm medial and 1–2 mm cranial to the midpoint of the lateral mass. It is oriented cranially by 25° laterally in the axial plane and parallel to the facet joints in the sagittal plane (approximately 20°–30°).^9^

Screws with diameter 3.5–4 mm, length 14–16 mm were preferred. In pedicle screwing, it approaches slightly lateral to the midpoint of the lateral mass and inferior to the inferior articular process of the adjacent superior vertebra. The 2.5 mm diamond drill was advanced approximately 35–45° medially and parallel to the endplate, then the tap was made and the screws were inserted.^10^ In pedicle screws, a thickness of 3.5–4 mm and a length of 24–30 mm were used. In some cases, connectors were used for rod fixation in patients in whom we placed lateral mass in the cervical region and pedicle screws in the upper thoracic region.

### Statistical Methods

In this study, the paired *t*‐test method was used to analyze the changes in the TS‐CL angles of the patients after C2‐T2, C3‐C6, and Subaxial‐T2 stabilization surgeries. This test measures whether the mean difference between paired observations in the sample is statistically significant. In the analysis, a *p*‐value of <0.05 was considered statistically significant for differences between pre‐ and postoperative measurements.

Pearson correlation coefficient was used for correlation analysis. This coefficient takes values between +1 and −1, with +1 indicating a perfect positive correlation, −1 meaning a perfect negative correlation, and 0 indicating no correlation between the two variables.

The statistical analyses and visualizations were performed using Python programming language with the following libraries: Matplotlib (Version 3.4.3; Python Software Foundation, USA) for creating the graphs and visualizations, NumPy (Version 1.21.2; Python Software Foundation, USA) for numerical computations and data manipulation.^11^, ^12^

## Results

### General Results

Average ages for each group are as follows:

Average age for C2‐T2 group: 63.78 years (±9.80).

Average age for C3‐C6 group: 60.30 years (±8.84).

Average age for the Subaxial‐T2 group: 61.42 years (±6.89).

The total mean age of the three groups is approximately 61.27 years.

**FIGURE 1:**
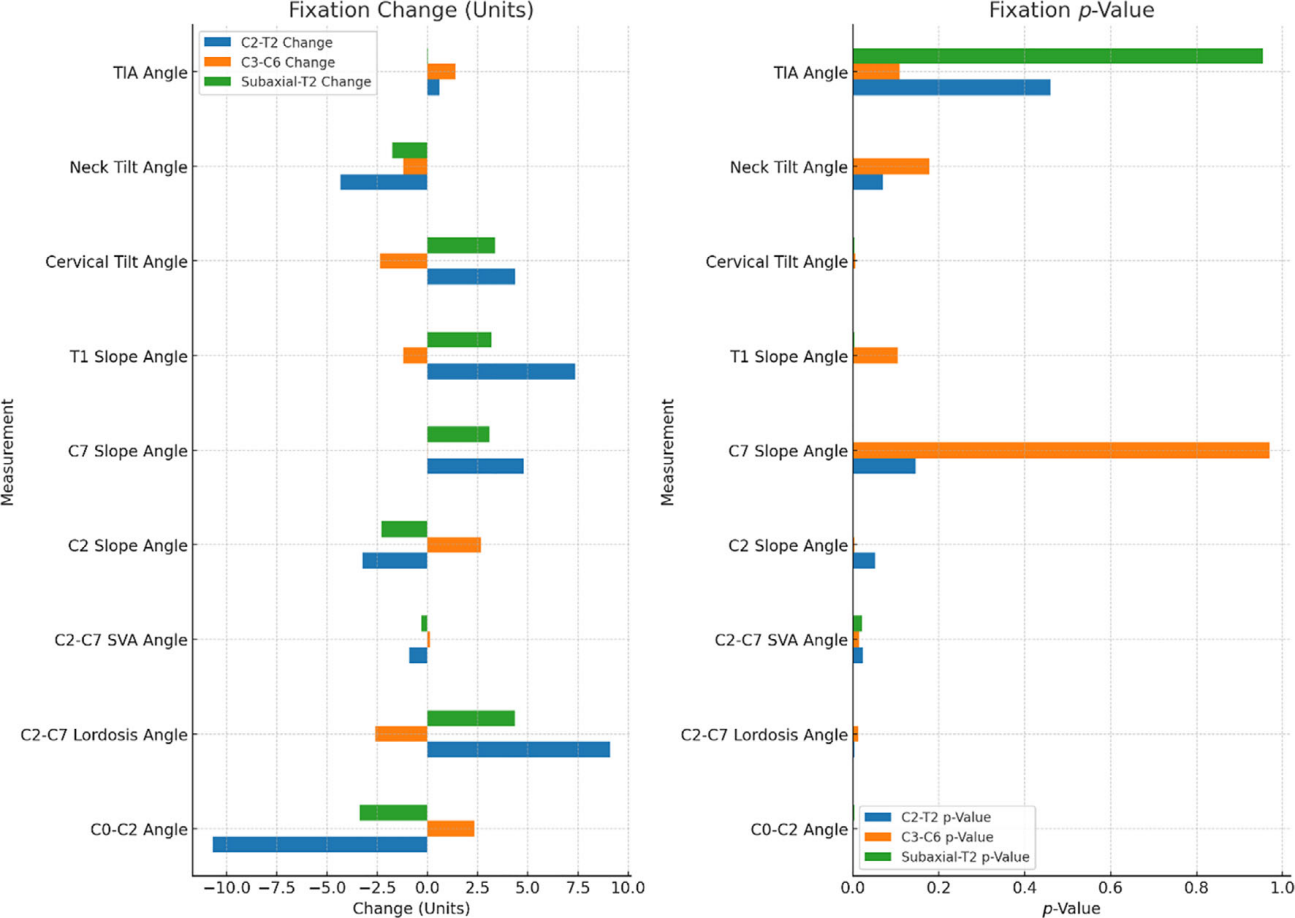
Angle changes and *p*‐values across different surgical groups. This figure illustrates the changes in various spinal angles and the statistical significance of these changes across C2‐T2, C3‐C6, and Subaxial‐T2 surgical groups. The left panel shows the mean changes in different spinal angles (units) for each group. The right panel displays the p‐values of these changes, indicating their statistical significance (C2‐T2: Blue; C3‐C6: Orange; Subaxial‐T2: Green).

Here's the corrected table that combines the changes and *p*‐values for each measurement across the three surgical techniques (C2‐T2, C3‐C6, and Subaxial‐T2) (Table 1, Figure 1).

**TABLE 1:** The change in each measured angle before and after the surgery, along with the statistical significance of these changes for each surgical technique.

Groups		C0‐C2 angle	C2‐C7 Lordosis angle	C2‐C7 SVA angle	C2 Slope angle	C7 Slope angle	T1 Slope angle	Cervical tilt angle	Neck tilt angle	TIA
C2‐T2	Change	−10.70	+9.12	−0.89	−3.22	+4.80	+7.37	+4.37	−4.33	+0.61
	*p*‐value	0.00	0.00	0.02	0.05	0.14	0.00	0.00	0.07	0.45
C3‐C6	Change	+2.36	−2.60	+0.14	+2.67	−0.03	−1.21	−2.36	−1.20	+1.39
	*p*‐value	0.00	0.01	0.03	−0.00	0.97	0.10	0.00	0.17	0.10
Subaxial‐T2	Change	−3.36	+4.36	−0.30	−2.28	+3.09	+3.20	+3.36	−1.75	+0.03
	*p*‐value	0.00	0.00	0.02	0.00	0.00	0.00	0.00	0.00	0.95

Abbreviation: TIA, thoracic inlet angle.

### Radiographic Indicators

**FIGURE 2:**
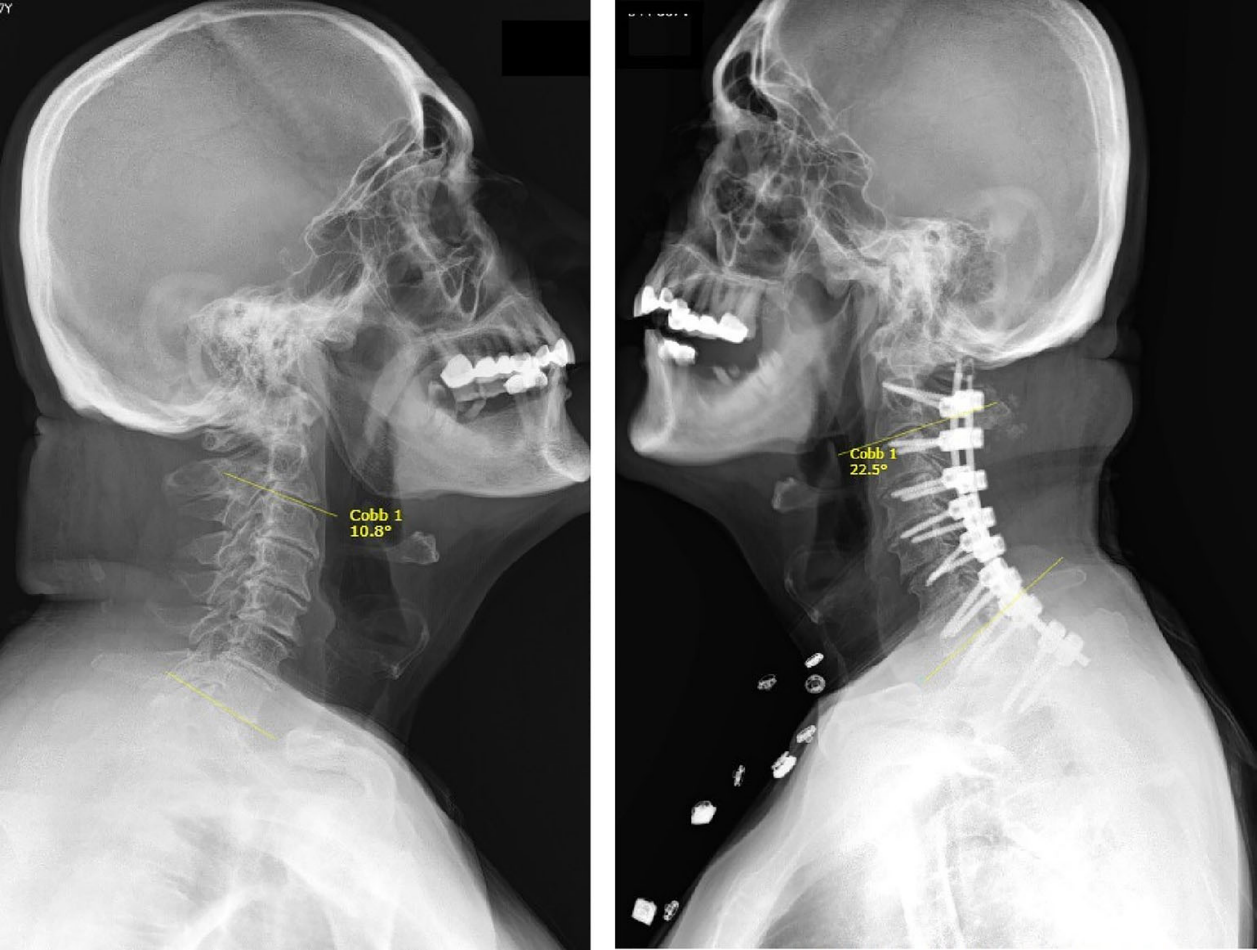
Preoperative and postoperative images of C2‐T2 stabilization. Cobb angles formed by yellow lines drawn from the lower ends of C2 and C7 are observed.

In performing C2‐T2 surgeries, we observed wide changes in measurements such as C0‐C2 angle and T1 slope angle and these changes were found to be statistically significant (*p* < 0.05). This result indicates that C2‐T2 surgery has a significant effect on these measurements. C2‐C7 lordosis angle and cervical tilt angle also increased significantly and these increases were found to be significant, indicating that surgery increases lordosis in these areas and affects cervical tilt (Figure 2). Neck tilt angle and TIA changes are not statistically significant, this shows that surgery did not have a significant effect on these measurements. For C2 slope angle, the change observed in the C2‐T2 surgical method was negative (−3.22) and this change was borderline statistically significant (*p* = 0.05). According to the paired *t*‐test result evaluating the change in TS‐CL angle, the t‐statistic was measured as 3.21 and the p‐value was 0.012. This result shows that the p‐value is less than 0.05, which means that the change between TS‐CL angles before and after surgery is statistically significant. Right C5 paralysis developed in one patient in this surgical group. Physical rehabilitation started instantly. At the end of the 2nd month, a near‐total improvement in abduction restriction was observed.

**FIGURE 3:**
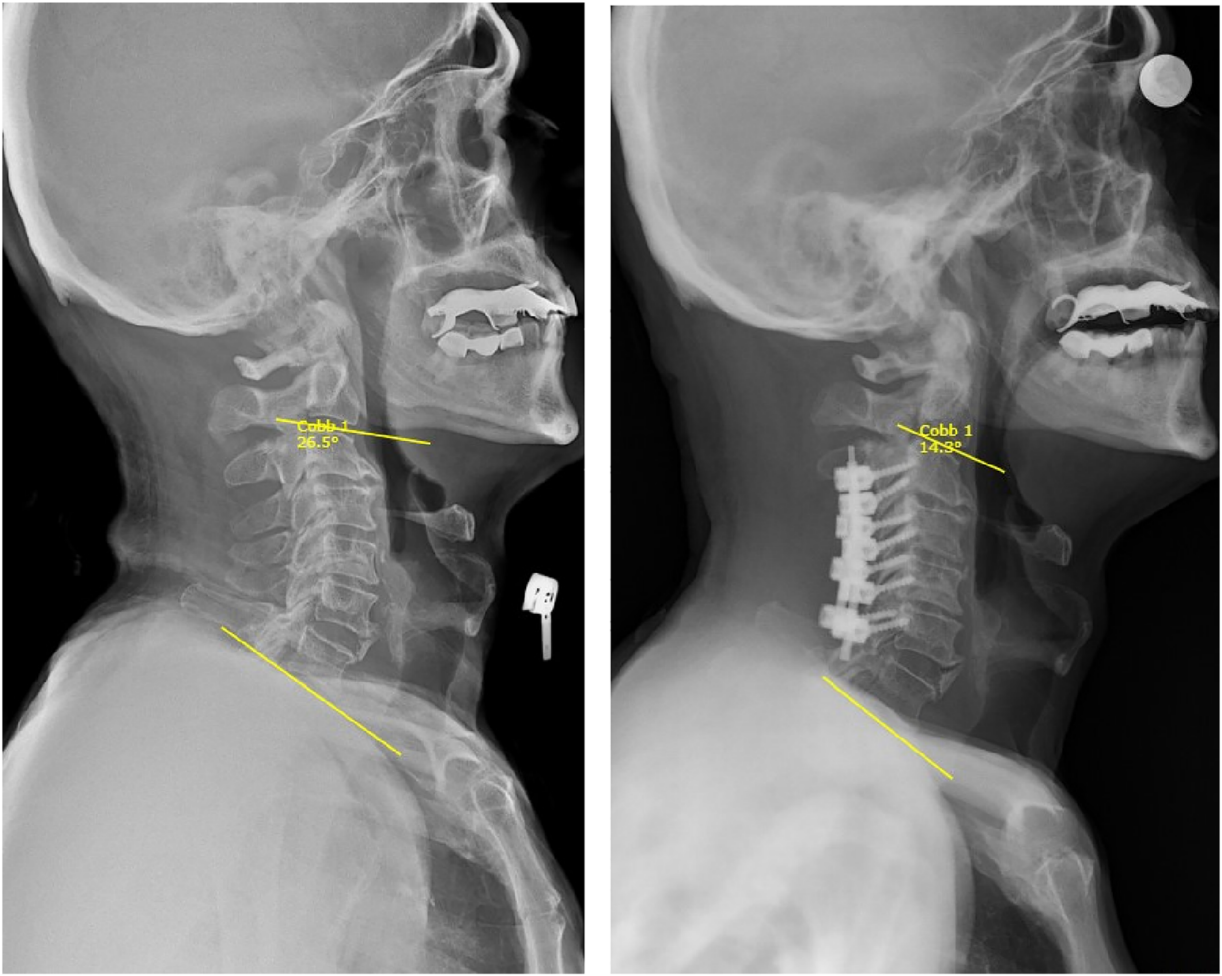
Preoperative and postoperative images of patient operated with lateral mass technique. Cobb angles formed by yellow lines drawn from the lower ends of C2 and C7 are observed.

In performing C3‐C6 surgeries, we observed that C0‐C2 angle and neck tilt angle changes were positive and statistically significant, verifying that C3‐C6 surgery increased these angles. C2‐C7 lordosis angle and cervical tilt angle changes are negative and these changes are statistically significant. This indicates that surgery has a reducing effect on lordosis and cervical tilt (Figure 3). Changes in C7 slope angle and TIA were not statistically significant, indicating no significant impact on these measurements. The average change in C2 slope angle before and after surgery increased by approximately 2.67° and this change was found to be statistically significant (*p* = 0.00). This result shows that the surgery caused a significant change in the C2 slope angle. According to the paired *t*‐test result evaluating the change in TS‐CL angle in patients undergoing C3‐C6 stabilization surgery, the t‐statistic was measured as −2.48 and the p‐value was 0.020. This result shows that the *p*‐value is less than 0.05, which means that the change between TS‐CL angles before and after surgery is statistically significant. No complications occurred in the postoperative period in this surgical group.

**FIGURE 4:**
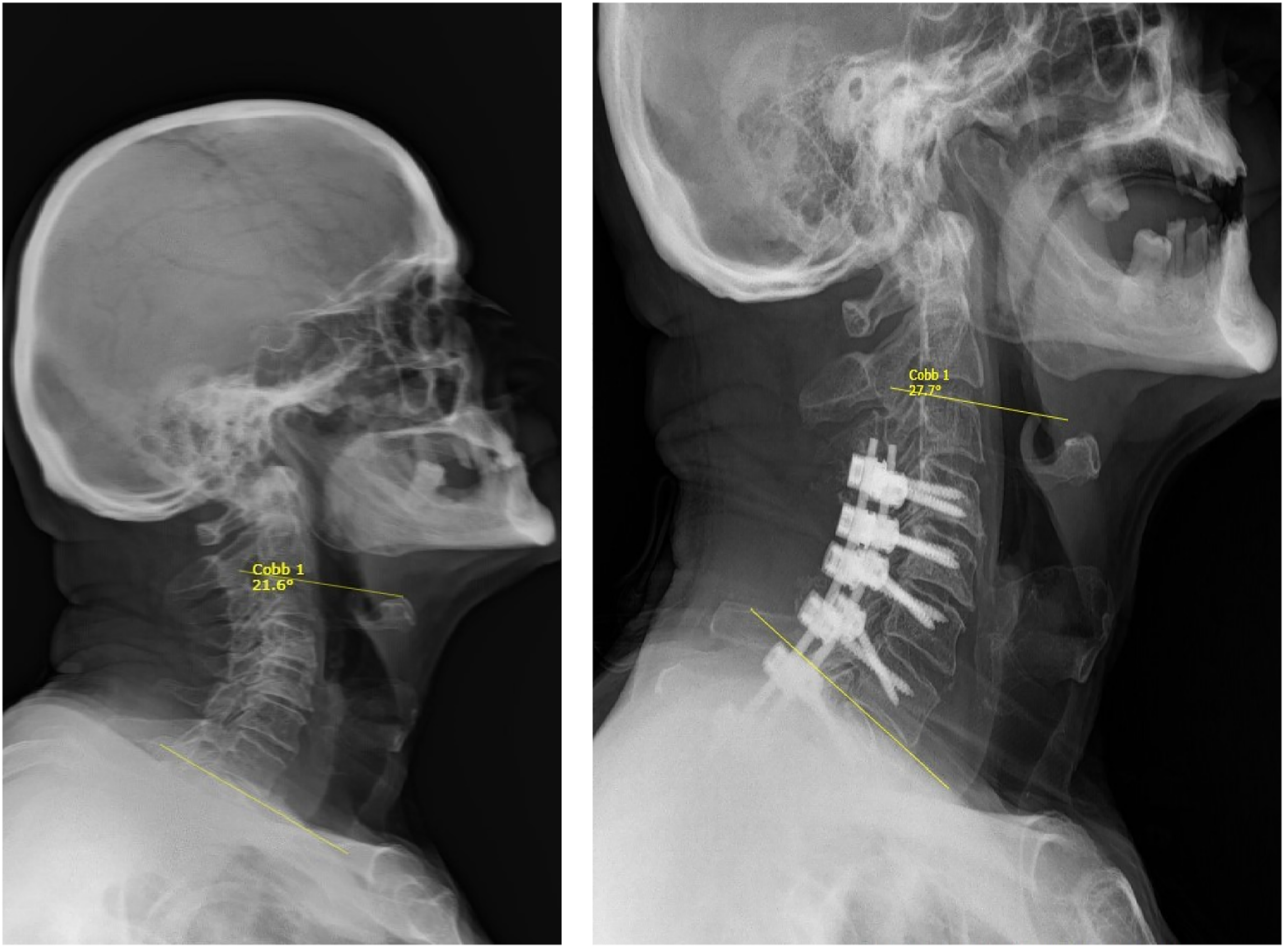
Preoperative and postoperative images of subaxial‐thoracal stabilization. Cobb angles formed by yellow lines drawn from the lower ends of C2 and C7 are observed.

Subaxial‐T2 surgical method showed the changes in C2‐C7 lordosis angle and C7 slope angle were positive and these changes were statistically significant. This indicates that Subaxial‐T2 surgery increases lordosis and C7 slope (Figure 4). C0‐C2 angle changes are negative and these changes are significant, verifying that the surgical method reduces the C0‐C2 angle. TIA change is not statistically significant, indicating that surgery did not have a significant effect on this measure. The change observed in the Subaxial‐T2 surgical method for C2 slope angle is negative (−2.28) and this change is statistically significant (*p* = 0.00). This indicates that Subaxial‐T2 surgical intervention also reduces the C2 tilt angle. According to the paired t‐test result evaluating the change in TS‐CL angle in patients who underwent subaxial‐T2 stabilization surgery, the t‐statistic was measured as 1.37 and the p‐value was 0.183. This result indicates that the p‐value is greater than 0.05, which means that the change between TS‐CL angles before and after surgery is not statistically significant. Superficial infection developed in one patient in this surgical group. Following the recommendations of the infectious diseases department, he recovered with ampicillin‐sulbactam treatment. No debridement was needed.

#### TS‐CL Angle–C2 Slop Angle Correlation

In the correlation made with Pearson coefficient correlation, a strong correlation was observed between the preop TS‐CL angle and the preop C2 slop angle, with a value of 0.78, and between the postop TS‐CL angle and the postop C2 slop angle, with a value of 0.85.

#### C7slop Angle–T1 Slop Angle Correlation

The correlation coefficient between preoperative C7 and T1 slope angles for the three surgical methods was measured to be approximately 0.73, indicating a strong positive correlation. The correlation coefficient between postoperative C7 and T1 slope angles was found to be approximately 0.70, verifying a strong positive correlation.

### Clinical Outcome

Here is the table showing the average changes in NDI and VAS scores for the three surgical groups (Table 2).

**TABLE 2:** The average preoperative and postoperative 3 months, 6 months, and 1‐year NDI and VAS scores for each surgical group.

Groups	Preop NDI mean	3 month NDI mean	6 month NDI mean	1 year NDI mean	Preop VAS mean	3 month VAS mean	6 month VAS mean	1 year VAS mean
Subaxial‐T2	35.33	32.79	31.38	29.13	28.38	26.54	24.63	22.75
C3‐C6	25.74	22.74	28.74	35.37	22.41	21.04	25.41	30.78
C2‐T2	35.33	30.11	25.78	19.89	27.89	23.11	20.89	16.67

Abbreviations: NDI, neck disability index; VAS, visual analog scale.

C2‐T2 surgery group shows a significant decrease in both NDI and VAS scores in time, indicating that surgical intervention led to significant improvements in these patients' neck function and pain levels.

C3‐C6 surgery group revealed an increase in NDI scores in time and a decrease is first observed in VAS scores and then an increase is observed at the end of 1 year. The increase in NDI indicates that the inhibition of neck functions increases in time.

Subaxial‐T2 surgery group revealed a significant decrease in both NDI and VAS scores in time. This reduction suggests that surgical intervention reduces pain and interference with patients' neck functions.

## Discussion

It is strongly recommended to evaluate the cervical sagittal alignment before posterior cervical surgeries and restore it in the perioperative period.^13^ Preoperative cervical sagittal imbalance is directly related to poor functional results after surgery.^14^

Posterior stabilization surgery is widely used in the treatment of cervical degenerative diseases. The most commonly used method is the lateral mass screwing technique, which has a shorter learning curve and fewer risks and was first described by Roy‐Camille^15^ and later modified by Magerl^9^ and Anderson.^16^ Another method is the subaxial pedicular screwing technique described by Abumi et al.^17^

In this study, we compared the cervical sagittal alignments of patient groups to whom we performed three different surgical fixations with two different techniques due to cervical degenerative disorder.

### Effects of C3‐C6, Subaxial‐T2, and C2‐T2 Groups on C2‐C7 Lordosis Angle

C2‐C7 lordosis angle was reported as 13.9° ± 12.3° by Yukawa et al.^18^ in the asymptomatic population and this angle was found to be 23° by Gore et al.^19^ in the asymptomatic population. In our study, it was observed that there was a change of +9.12 in the C2‐T2 group, +4.36 in the subaxial‐T2 group, and −2.60 in the C3‐C6 group. We observed that the C2‐T2 surgery group was significantly more effective in correcting the C2‐C7 lordosis angle, while the C3‐C6 surgery group had a significantly negative effect.

### Effects of C3‐C6, Subaxial‐T2, and C2‐T2 Groups on C2‐C7 SVA

C2‐C7 SVA was shown to be significantly associated with Japanese Orthopaedic Association (mJOA) scores in patients with cervical spondylopathic myelopathy.^20^ In another study, it was stated that there was a positive correlation with neck disability index (NDI) scores and a negative correlation with SF‐36 physical component scores, and that C2‐C7 SVA greater than 40 mm negatively affected quality of life outcomes.^21^ In our study, it was observed that the C2‐T2 group was superior to the subaxial‐T2 group in correcting the C2‐C7 SVA disorder. It was observed that the C3‐C6 group had a negative effect.

### Effects of C3‐C6, Subaxial‐T2, and C2‐T2 Groups on C0‐C2 Angle

As cervical lordosis decreases, the C0‐C2 angle increases, in other words, as the C2‐C7 lordosis angle decreases, the C0‐C2 angle increases.^22^ In our study, we saw that the C0‐C2 angle decreased significantly in the C2‐T2 group, significantly in the subaxial‐T2 group, and significantly in the C3‐C6 group. Our findings supported that it works inversely with the C0‐C2 angle and the C2‐C7 angle.

### Evaluation of the Correlation between C2 Slop Angle and TS‐CL Angles

The relationship between C2S and TS‐CL has been investigated with various parameters and it has been shown that there is harmony between them. For this reason, there are researchers who accept the C2S angle directly instead of the TS‐CL angle.^23^ Also Chai et al., in their recent study, Al suggested evaluating the tendency of cervical alignment in cervical degenerative disorders through the C2 slope angle. They revealed that C2S correlated with both cervical alignment and HRQOL.^24^ In the correlation test we conducted and we observed a very strong correlation between the TS‐CL angle and the C2S angle, such as 0.78 preoperatively and 0.85 postoperatively.

### Evaluation of the Correlation between C7 Slop Ile and T1 Slop Angle

Tamai et al.^25^ suggested in their study with kinetic MRI images that there was a strong correlation between C7 slop and T1 slop angle. Other studies conducted with kinetic Mr. and Bt images have stated that there is a correlation between T1 angle and C2‐C7 SVA and C2‐C7 lordosis angle.^26^, ^27^ In our correlation study, we also observed that there is a strong relation between C7 slop and T1 slop angle, similar to previous studies.

T1 slope angle is a major parameter used in the evaluation of preoperative and postoperative cervical alignment due to its correlation with C2‐C7 angle and SVA.^28^, ^29^ The positive correlation of T1 slope angle with cervical lordosis requires more cervical lordosis for larger T1 slope angle.^30^ TS‐CL angle is an angle we obtained by subtracting the C2‐C7 angle from the T1S angle. We observed that the TS‐CL angle decreased significantly in the patients we performed C3‐C6 lateral mass surgery, the TS‐CL angle increased but was not statistically significant in the surgery we performed with subaxial‐T2 pedicle screws, and the TS‐CL angle increased significantly in the surgery we performed with the C2‐T2 pedicle screw technique. This proved that it was superior to other techniques on lordosis angle according to preoperative evaluations in the C2‐T2 surgery group.

### Effects of C3‐C6, Subaxial‐T2, and C2‐T2 Groups on NDI and VAS

It has been reported that C2‐C7 SVA is positively correlated with NDI in patients followed after posterior cervical stabilization.^21^ We observed the best SVA values in the C2‐T2 group and the worst SVA values in the C3‐C6 group. We found the best NDI values in the C2‐T2 group, consistent with the SVA values. We think that the C2‐T2 group, in which we obtained better C2‐C7 lordosis and lower SVA at the end of 1 year, is a more appropriate choice both radiologically and clinically.

### Evaluation According to Surgical Margins

Heary et al.^13^ reported in their study with 56 cases, that they did not observe a significant difference in terms of lordosis angle between the fixations that ended at C7 and the fixations that ended higher. Mayfield stated that they used the title effectively. As a result of their study, they stated that they preserved cervical lordosis and corrected mild–moderate cervical kyphosis. We obtained a better C2‐C7 lordosis angle in the C2‐T2 group and Subaxial‐T2 group, in which we included C7 in the system, compared to the C3‐C6 group. We attribute the difference to the fact that the case series in Heary et al.'s study were not distributed homogeneously and that the Mayfield headgear did not allow us to provide lordosis due to the limited lateral joint movements between C3 and C6. We use the Mayfield headgear routinely to provide lordosis. We believe that when the upper limit increases to C2, the lateral joint movement between C2‐C3 is greater and it is effective in providing lordosis with the help of the Mayfield headgear, and keeping the C2 pedicle entry point higher than the subaxial region helps to increase lordosis with the help of the rod.

Lee et al.^31^ found early postoperative SVA to be significantly lower and cervical lordosis to be significantly higher in the CPS group in their series of 71 patients, including 51 cervical pedicular screwing (CPS) and 20 cervical lateral mass screwing (LM) surgeries. They stated that there was no significant difference between the CPS group and LM group SVA, T1S, and CL angles during an average follow‐up of 2–3 years. In our study, in the 1‐year follow‐up of the C2‐T2 and subaxial‐T2 groups, the lower SVA, higher CL, and higher T1 slop continued to be the same compared to the C3‐C6 group. We attribute the fact that Lee et al. did not include surgical groups containing more than five levels in his study, as can be seen from the images of the CPS group, some of them are subaxial, some of them extend to C2, and the lower end is not clearly defined.

Fixations performed with pedicle screws are more stable under flexion‐extension loading than fixations performed with lateral mass screws.^32^ Previous studies have stated that it may be necessary to perform additional anterior fusion to ensure cervical alignment and stability in stabilization with lateral mass in patients with cervical injury and cervical deformity.^33^, ^34^ In our study, we observed that the C2‐T2 lordosis angle improved and the SVA values decreased in the C2‐T2 and subaxial‐T2 groups in which we used pedicular screw fixation.

In this study, we retrospectively analyzed patient groups due to cervical degenerative disorder in two different ways and determined the starting and ending points in three different ways. We tried to reveal the effects of the surgery we perform using pedicular screws and the surgeries we perform using lateral mass screws on cervical alignment in clinic and practice.

### Limitation and Strengths

There were some limitations in our study. First, it is a single‐center retrospective study and secondly, the number of patients in the C2‐T2 group, in which we achieved the best results, was less than the other groups. Longer case series are needed to evaluate cervical alignment in cervical degenerative disorders covering the range C3‐C6, with the upper limit being C2 in the cervical region and the lower limit being T2 in the upper thoracic region.

When we examined other articles on this subject, the starting and ending points of fixation were clearly revealed in our study. Cervical region angles were examined in a wide range and some angles were correlated with each other. All of these data were included in the same study.

## Conclusion

In this study, we evaluated cervical alignment both radiologically and clinically in cervical degenerative disorders preoperatively and postoperatively. In pathologies affecting almost the entire cervical spinal canal from C2 or C3 to C6, we recommend fixation with C2‐T2 pedicular screws, where we achieve angles closest to our desired values. If the pathology begins below the C3 distance, we believe that subaxial‐T2 fixation is appropriate for cervical alignment.

## Conflict of Interest Statement

The authors declare no competing interests.

## Ethics Statement

This study was performed in line with the principles of the Declaration of Helsinki. Ethical approval was given by the Sakarya University Rectorate, Faculty of Medicine, Non‐Interventional Ethics Committee (23.02.2024/No. E‐71522473‐050.04‐340191‐44).

## Author Contributions

Supervision project administration: Mustafa Kaya and Davut Ceylan. Resources: Mustafa Kaya, Davut Ceylan, Tibet Kacira, Sabahattin Hiziroglu, and Cigdem Erdin. Conceptualization: Mustafa Kaya, Davut Ceylan, Tibet Kacira, Sabahattin Hiziroglu, and Cigdem Erdin. Methodology: Mustafa Kaya, Davut Ceylan, and Tibet Kacira. Data Curation: Mustafa Kaya, Davut Ceylan, Tibet Kacira, and Özlem Kitiki Kacira. Writing–review & editing: Mustafa Kaya, Davut Ceylan, Tibet Kacira, Özlem Kitiki Kacira, Cigdem Erdin, and Sabahattin Hiziroglu. Writing Original Draft: Mustafa Kaya and Tibet Kacira. Software: Mustafa Kaya and Özlem Kitiki Kacira. Validation: Mustafa Kaya and Tibet Kacira.

## Funding Information

This research received no specific grant from any funding agency in the public, commercial, or not‐for‐profit sectors.
